# Additive predictive value of triglyceride-glucose index and epicardial adipose tissue volume for major adverse cardiovascular events following coronary artery bypass grafting

**DOI:** 10.3389/fendo.2025.1730404

**Published:** 2026-01-14

**Authors:** Juan Wang, Run Zhang, Zhihui Yan, Shimiao Ruan, Jia Liu, Zhengliang Li, Fangfang Shang, Wenzhong Zhang

**Affiliations:** 1 Department of Cardiology, The Affiliated Hospital of Qingdao University, Qingdao, Shandong, China; 2 Department of Cardiology, Linzi District Maternal and Child Health Hospital (Qidu Hospital), Zibo, Shandong, China; 3 Department of Cardiology, Laizhou People’s Hospital, Yantai, Shandong, China; 4 Department of Pathology, Navy 971 Hospital, Qingdao, Shandong, China

**Keywords:** coronary artery bypass grafting, epicardial adipose tissue, interaction, major adverse cardiovascular events, risk prediction, triglyceride-glucose index

## Abstract

**Background:**

The triglyceride-glucose (TyG) index is a simple and reliable marker of insulin resistance and is associated with cardiovascular risk. Epicardial adipose tissue (EAT) volume reflects local visceral fat burden and also correlates with cardiovascular events. While both markers have been studied individually, their combined predictive value for major adverse cardiovascular events (MACE) after coronary artery bypass grafting (CABG) remains unclear. This study evaluated whether TyG index and EAT volume, alone or in combination, can improve risk prediction of MACE following CABG and assessed their potential interaction.

**Methods:**

We retrospectively analyzed 304 patients who underwent CABG between 2018 and 2022. TyG index and EAT volume were measured preoperatively. Patients were stratified based on optimal cut-off values derived from ROC analysis. Cox regression models were used to estimate associations with MACE. Interaction was assessed using relative excess risk due to interaction (RERI), attributable proportion (AP), and synergy index (SI). Model performance was evaluated using C-statistic, net reclassification improvement (NRI), and integrated discrimination improvement (IDI). Model fit was assessed with the Akaike information criterion (AIC), Bayesian information criterion (BIC).

**Results:**

During follow-up of 44 months, 82 patients experienced MACE. Both TyG index and EAT volume were independently associated with increased risk. Patients with elevations in both markers had a significantly higher risk (adjusted HR = 7.62, 95% CI: 3.27–17.76). A significant additive interaction was observed (RERI = 3.81; AP = 0.50; SI = 2.34). Adding both variables improved model discrimination and fit.

**Conclusion:**

TyG index and EAT volume are independent predictors of MACE after CABG. Their combined assessment provides additional information for risk stratification, but the findings are preliminary and require validation in larger, prospective, multi-center studies.

## 1 Introduction

Coronary artery disease (CAD) remains a major cause of death worldwide despite advances in diagnosis and treatment (1). Coronary artery bypass grafting (CABG) is the preferred revascularization method for patients with complex multivessel disease, left main coronary disease, diabetes, or reduced ventricular function (2, 3). However, long-term outcomes after CABG remain suboptimal, and better risk stratification is needed (4, 5).

Insulin resistance (IR) is common in CAD and contributes to the progression of atherosclerosis. The triglyceride–glucose (TyG) index, calculated from fasting glucose and triglyceride levels, is a simple and reliable surrogate marker of IR and has been widely associated with cardio-metabolic disorders (6, 7). Studies have shown that a higher TyG index is positively associated with adverse outcomes in CAD patients, particularly after revascularization procedures (8–10). Recent studies have extended the clinical relevance of IR to surgical populations, showing that TyG is associated with long-term cardiovascular risk in patients undergoing CABG, and a large cohort study further confirmed that TyG is a superior IR marker for predicting long-term major adverse cardiovascular events after CABG (11, 12).Epicardial adipose tissue (EAT), a visceral fat depot around the heart, promotes inflammation and metabolic stress, and higher EAT volume has been associated with adverse cardiac events (13–17).

Previous studies have evaluated TyG index and EAT separately, but few have examined their combined effect on prognosis after CABG. IR and EAT may interact through inflammatory and metabolic pathways, potentially amplifying cardiovascular risk (18, 19).

This study aimed to evaluate the independent and combined predictive value of TyG index and EAT volume for major adverse cardiovascular events (MACE) after CABG, and to explore whether their combination improves risk prediction compared with each marker alone.

## 2 Article types

Original Research

## 3 Methods

### 3.1 Study population

We retrospectively analyzed 304 patients who underwent CABG at the Affiliated Hospital of Qingdao University from January 2018 to December 2022. Telephone follow-up was conducted in November to December 2024. The median follow-up duration was 44 months (IQR 31–62 months), during which 82 patients experienced MACE.

Inclusion criteria: age ≥18 years, preoperative CCTA during hospitalization, complete medical records, and ability to complete follow-up.

Exclusion criteria: concomitant surgery (valve surgery, surgical ablation, or congenital heart surgery); prior PCI or CABG; severe cardiomyopathy (LVEF <40% or severe left ventricular dilation [LVEDD >65 mm]); severe pulmonary, hepatic (Child-Pugh B/C), or renal disease (eGFR <30 mL/min/1.73 m²); malignancy; severely elevated triglycerides (TG ≥5.65 mmol/L) or suspected familial hypertriglyceridemia; or missing key data.

This was a non-interventional, retrospective study approved by the institutional ethics committee and conducted in accordance with the Declaration of Helsinki.

### 3.2 Data collection

Clinical data were obtained from the hospital’s electronic medical record system, including demographic characteristics, medical history, comorbidities, and medication use. Venous blood samples were collected after overnight fasting (from 10:00 PM the previous night) at approximately 6:00 AM on the day following hospital admission, prior to surgery. Fasting plasma glucose (FPG), serum creatinine (SCr), and lipid profiles were measured according to standard hospital laboratory procedures.

Multivessel disease was defined as two- or three-vessel coronary artery involvement, and left main disease as ≥50% stenosis in the left main coronary artery, as assessed by preoperative coronary computed tomography angiography (CCTA). Hypertension was defined as systolic blood pressure (SBP) ≥ 140 mmHg, diastolic blood pressure (DBP) ≥ 90 mmHg, or use of antihypertensive. Diabetes was defined as random glucose ≥ 11.1 mmol/L, FPG ≥7.0 mmol/L, or use of hypoglycemic medications. Hyperlipidemia was defined as the use of total lipid-lowering agents or a total cholesterol ≥ 240 mg/dL or use of lipid-lowing therapy (20).

The TyG index was calculated as Ln [fasting TG (mg/dL) × FPG (mg/dL)/2], as originally described by Guerrero-Romero et al. (2010) (21). Epicardial adipose tissue (EAT) volume was quantified from coronary computed tomography angiography (CCTA) images using dedicated post-processing software (AWS). Two radiologists independently performed the measurements, with any discrepancies resolved by a senior radiologist. CCTA was conducted on a 128-slice dual-source cardiac CT scanner with acquisition parameters of 100 kV, 601 mA, and a slice thickness of 0.66 mm. Imaging covered the region from the aortic root to the cardiac apex. Three-dimensional reconstruction was performed and EAT volume was automatically calculated by the software.

MACE included all-cause mortality, repeat coronary revascularization, heart failure, severe arrhythmias, and stroke. Repeat revascularization was defined as subsequent percutaneous coronary intervention (PCI) or CABG of the index lesion. Heart failure was defined as impaired cardiac function due to myocardial ischemia, including cases where new-onset atrial fibrillation led to heart failure exacerbation requiring hospitalization. Severe arrhythmias included sustained ventricular tachycardia, high-grade atrioventricular block, and clinically significant sinus bradycardia (heart rate <40 bpm or requiring intervention). Stroke was defined as ischemic stroke due to interruption of cerebral blood flow; no hemorrhagic stroke events occurred in this cohort.

### 3.3 Statistical analysis

Statistical analyses were performed using SPSS 29.0 and R 4.4.2. A two-tailed *p*-value < 0.05 was considered statistically significant.

Receiver operating characteristic (ROC) curve was used to determine optimal cut-off values for the TyG index and EAT volume in predicting MACE. The optimal cut-offs were 8.65 for TyG (AUC = 0.624, 95% CI: 0.554–0.694, *p* < 0.05) and 116.2 for EAT (AUC = 0.586, 95% CI: 0.510–0.661, *p* < 0.05). Based on these cut-offs, patients were stratified into four groups: Group 1, low TyG and low EAT (TyG ≤ 8.65, EAT ≤ 116.2); Group 2, low TyG and high EAT (TyG ≤ 8.65, EAT > 116.2); Group 3, high TyG and low EAT (TyG > 8.65, EAT ≤ 116.2); and Group 4, high TyG and high EAT (TyG > 8.65, EAT > 116.2).

Baseline characteristics were compared across groups. Normality of continuous variables was evaluated using the Shapiro–Wilk test in SPSS, and all variables were determined to be non-normally distributed. Consequently, continuous variables were summarized as median (IQR) and compared using appropriate non-parametric tests (Mann–Whitney U or Kruskal–Wallis). Categorical variables were reported as counts (percentages) and compared using the chi-square test. Because no variable met the assumption of normality, assessment of variance homogeneity was not applicable. Potential outliers were assessed through boxplot visualization, and as no observations were indicative of measurement or data-entry errors, all data points were retained for analysis. Kaplan–Meier curves were generated to compare cumulative event rates among groups, with log-rank tests used for survival differences.

Cox proportional hazards regression was used to evaluate associations of TyG index and EAT volume with MACE. Model 1 adjusted for age and sex; Model 2 included variables with P < 0.05 in univariate analysis; Model 3 was further adjusted for all variables in Model 2 plus age, sex, left main disease, triglyceride level, and serum creatinine, based on penalized variable selection and collinearity assessment. To reduce model complexity and minimize the risk of overfitting, multicollinearity among candidate variables was assessed using variance inflation factors (VIFs), and least absolute shrinkage and selection operator (LASSO) regression was applied as a penalized variable selection approach before fitting the multivariable Cox model (22). TyG index and EAT volume were first analyzed as continuous variables, standardized into z-scores to reflect the effect per 1 SD increase and reduce outlier influence. They were also analyzed as categorical variables based on ROC cut-offs. Additive interaction between TyG index and EAT volume was assessed using RERI, AP, and SI (23–25). The SI represents a measure of statistical additive interaction; it should not be interpreted as indicating a mechanistic or biological synergistic effect. Incremental predictive value was evaluated with the C-statistic, net reclassification improvement (NRI), and integrated discrimination improvement (IDI), and model fit was assessed using the AIC, BIC, and the likelihood ratio test.

## 4 Results

### 4.1 Baseline characteristics of participants

ROC curve analysis identified an optimal TyG index cut-off of 8.65 for predicting MACE, based on the maximum Youden index. Baseline characteristics stratified by this cut-off are presented in **Table 1**. Of the 304 patients, those with TyG > 8.65 were more often female, had lower mean age, and exhibited higher BMI, FPG, TG, TC, and LDL-C, but lower HDL-C and EAT volume. They also had a higher prevalence of diabetes, greater use of glucose-lowering drugs, and an increased risk of MACE.

**Table 1:** Baseline characteristics of the study population stratified by TyG index (cut-off 8.65).

Variables	All (n=304)	TyG ≤ 8.65 (n=180)	TyG>8.65 (n=124)	*P* -value
General conditions				
Age (years)	64 (57,68)	65 (58,68)	63 (56,67)	0.034
Male, n (%)	226 (74.1)	143 (79.4)	83 (66.9)	0.014
BMI (kg/m2)	25.61 (23.66,27.68)	24.99 (23.47,27.43)	26.06 (24.22,28.34)	0.001
LVEF (%)	60 (57,63)	60 (57,62)	60 (55,63)	0.415
Left main disease, n (%)	105 (34.5)	65 (36.1)	40 (32.3)	0.487
Multivessel disease, n (%)	299 (98.4)	176 (97.8)	123 (99.2)	0.621
Risk factors, n (%)				
Current smoking	109 (35.9)	61 (33.9)	48 (38.7)	0.389
Current drinking	49 (16.1)	27 (15.0)	22 (17.7)	0.523
Hypertension	190 (62.5)	106 (58.9)	84 (67.7)	0.117
DM	105 (34.5)	44 (24.4)	61 (49.2)	<0.001
Hyperlipidemia	149 (49.0)	81 (45.0)	68 (54.8)	0.092
Laboratory tests				
FPG (mmol/L)	5.45 (4.98,6.35)	5.23 (4.82,5.62)	6.39 (5.43,8.82)	<0.001
TG (mmol/L)	1.26 (0.93,1.73)	1.00 (0.84,1.22)	1.94 (1.54,2.46)	<0.001
TC (mmol/L)	4.13 (3.33, 5.23)	3.97 (3.18,4.94)	4.49 (3.54,5.67)	<0.001
LDL-C (mmol/L)	2.43 (1.76,3.20)	2.27 (1.68,3.01)	2.80 (1.99,3.45)	<0.001
HDL-C (mmol/L)	1.15 (0.95,1.36)	1.21 (0.98,1.41)	1.10 (0.95,1.27)	0.008
SCr (μmol/L)	85.80 (72.00,98.00)	85.50 (72.00,97.68)	86.00 (71.00,99.45)	0.682
Imaging examination				
EAT (cm³)	117.4 (94.4,126.0)	121.1 (96.1,127.1)	106.6 (92.5,125.8)	<0.001
Medications at the time of discharge, n (%)				
Antiplatelet drugs	302 (99.3)	179 (99.4)	123 (99.2)	1.000
Statins	135 (44.4)	75 (41.7)	60 (48.4)	0.246
Hypoglycemic drugs	105 (34.5)	42 (23.3)	63 (50.8)	<0.001
**MACE, n (%)**	82 (27.0)	27 (15.0)	55 (44.4)	<0.001

TyG index triglyceride-glucose index, BMI body mass index, LVEF left ventricle ejection fraction, DM diabetes mellitus, FPG fasting plasma glucose, TC total cholesterol, TG triglyceride, LDL-C low-density lipoprotein-cholesterol, HDL-C high-density lipoprotein-cholesterol, SCr serum creatinine, EAT epicardial adipose tissue, MACE major adverse cardiovascular event.

Patients were also stratified by the optimal EAT volume cut-off (**Table 2**). Compared with those with lower EAT volume, patients with higher EAT volume showed a similar age distribution and a lower proportion of males. They had higher FPG and TG levels and were more likely to experience MACE.

**Table 2:** Baseline characteristics of the study population stratified by EAT volume (cut-off 116.2 cm ³).

Variables	EAT>116.2 (n=156)	EAT ≤ 116.2 (n=148)	*P* -valve
General conditions			
Age (years)	64 (57,69)	63 (57,67)	0.546
Male, n (%)	124 (79.5)	102 (68.9)	0.035
BMI (kg/m2)	25.43 (23.55,27.67)	25.81 (23.91,27.78)	0.346
LVEF (%)	60 (56,62)	61 (58,63)	0.176
Left main disease, n (%)	56 (35.9)	49 (33.1)	0.609
Multivessel disease, n (%)	155 (99.4)	144 (97.3)	0.336
Risk factors, n (%)			
Current smoking	58 (37.2)	51 (34.5)	0.621
Current drinking	24 (15.4)	25 (16.9)	0.721
Hypertension	99 (63.5)	91 (61.5)	0.722
DM	49 (31.4)	56 (37.8)	0.239
Hyperlipidemia	78 (50.0)	71 (48.0)	0.724
Laboratory tests			
FPG (mmol/L)	5.61 (5.14,6.87)	5.38 (4.79,6.12)	<0.001
TG (mmol/L)	1.35 (1.16,1.93)	1.02 (0.80,1.57)	<0.001
TC (mmol/L)	4.17 (3.37,5.35)	4.09 (3.20,5.12)	0.406
LDL-C (mmol/L)	2.48 (1.84,3.32)	2.32 (1.72,3.13)	0.304
HDL-C (mmol/L)	1.14 (0.96,1.36)	1.16 (0.95,1.36)	0.819
SCr (μmol/L)	84.00 (69.25,95.98)	88.00 (73.25,100.60)	0.063
Medications at the time of discharge, n (%)			
Antiplatelet drugs	155 (99.4)	147 (99.3)	1.000
Statins	68 (43.6)	67 (45.3)	0.768
Hypoglycemic drugs	49 (31.4)	56 (37.8)	0.239
MACE, n (%)	57 (36.5)	25 (16.9)	<0.001

TyG index triglyceride-glucose index, BMI body mass index, LVEF left ventricle ejection fraction, DM diabetes mellitus, FPG fasting plasma glucose, TC total cholesterol, TG triglyceride, LDL-C low-density lipoprotein-cholesterol, HDL-C high-density lipoprotein-cholesterol, SCr serum creatinine, EAT epicardial adipose tissue, MACE major adverse cardiovascular event.

### 4.2 Predictive value of TyG index and EAT volume for MACE

During a median follow-up of 44 months (IQR31–62), 82 patients (27.0%) experienced MACE.5 (1.6%) all-cause mortality, 28 (9.2%) repeat revascularization, 10 (3.3%) of heart failure, 7 (2.3%) severe arrhythmias, and 36 (11.8%) nonfatal stroke. As some patients experienced multiple adverse events during follow-up, the summed counts of individual event categories exceed the total number of patients with MACE.

**Figure 1:**
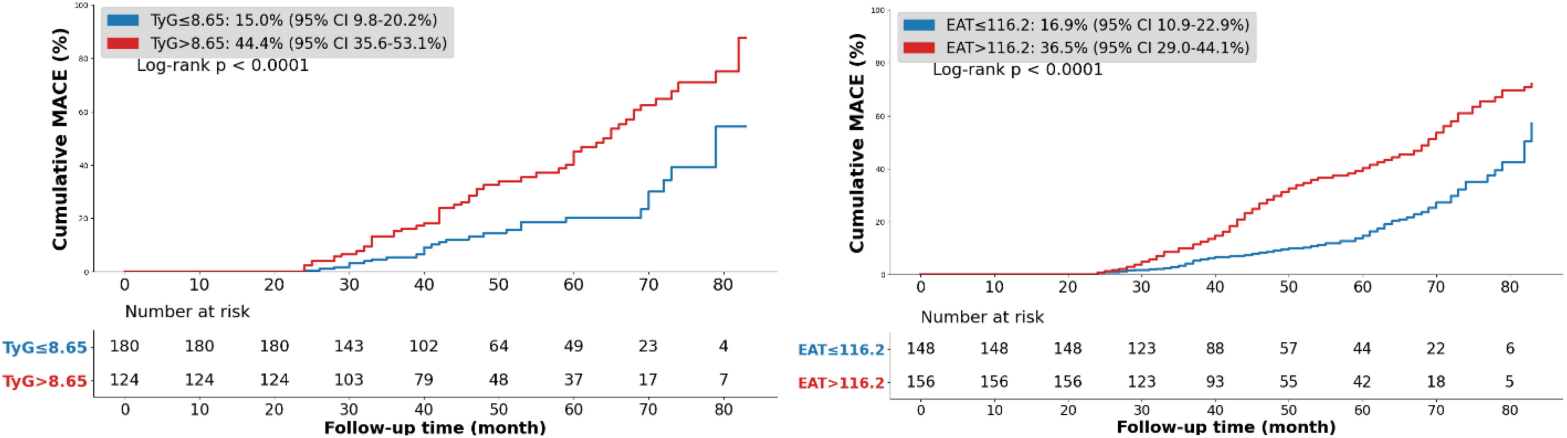
Kaplan–Meier survival curves according to the TyG index and EAT. TyG index triglyceride-glucose index, EAT epicardial adipose tissue, MACE major adverse cardiovascular event.

Kaplan–Meier showed significantly higher cumulative MACE incidence in patients with TyG > 8.65 compared with those ≤ 8.65 and in those with EAT > 116.2 compared with lower volumes (*p* < 0.0001, **Figure 1**.).

**Figure 2:**
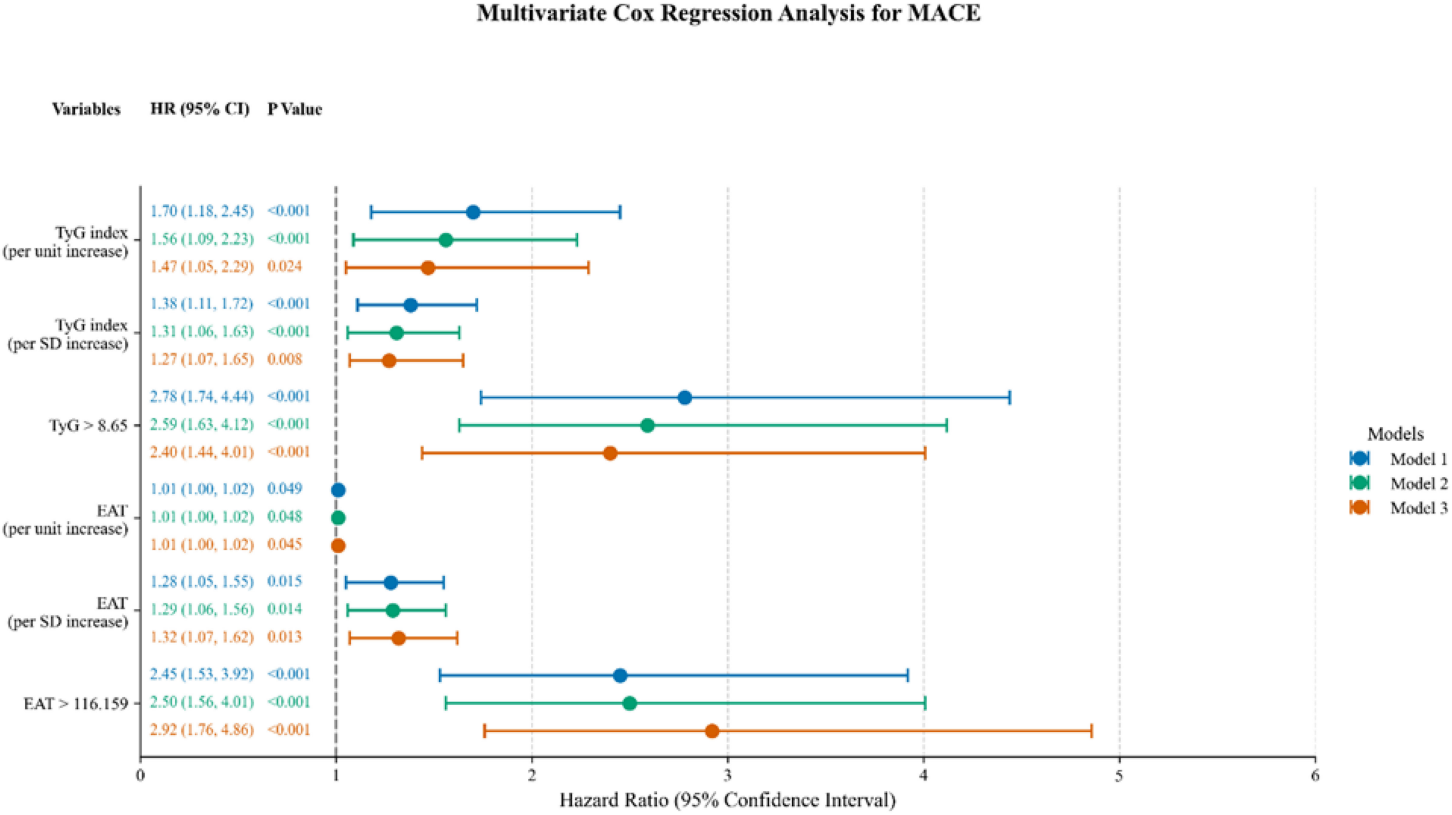
Multivariable Cox proportional hazards regression for MACE across three models. Model 1: adjusted for age and sex; Model 2: variables with p<0.05 in univariate analysis; Model 3: fully adjusted model. HR, hazard ratio; CI, confidence interval; TyG, triglyceride–glucose index; EAT, epicardial adipose tissue.

In univariate Cox regression, both TyG index and EAT volume were significantly associated with MACE; these associations persisted after adjustment for conventional cardiovascular risk factors (**Table 3**; **Figure 2**.). Compared with patients with low TyG/low EAT volume, high TyG was associated with a 2.40-fold increased risk of MACE, and high EAT with a 2.92-fold higher risk.

**Table 3:** Multivariate Cox regression analysis for MACE.

Variables	HR (95% CI)	Model 2	Model 3
	Model 1		
TyG index			
Per unit increase	1.70 (1.18–2.45) *	1.56 (1.09–2.23) *	1.47 (1.05–2.29) *
Per SD increase	1.38 (1.11–1.72) *	1.31 (1.06–1.63)*	1.27 (1.07–1.65)*
TyG ≤ 8.65	1 (ref)	1 (ref)	1 (ref)
TyG>8.65	2.78 (1.74–4.44)*	2.59 (1.63–4.12)*	2.27 (1.31–3.94)*
EAT			
Per unit increase	1.01 (1.00–1.02) *	1.01 (1.00–1.02) *	1.01 (1.00–1.02)*
Per SD increase	1.28 (1.05–1.55) *	1.29 (1.06–1.56)*	1.29 (1.07–1.55)*
EAT ≤ 116.2	1 (ref)	1 (ref)	1 (ref)
EAT>116.2	2.45 (1.53–3.92) *	2.50 (1.56–4.01)*	2.82 (1.74–4.59)*

Model 1: Adjusted for age and gender.

Model 2: Adjusted for variables with *P*-value < 0.05 in univariate analysis, including BMI, LVEF.

Model 3: Adjusted for all the variables in Model 2 plus age, gender, left main disease, triglyceride and serum creatinine.

TyG index triglyceride-glucose index, EAT epicardial adipose tissue, MACE major adverse cardiovascular events, Ref = reference (baseline) model.

*p < 0.05

### 4.3 Combined effect of TyG index and EAT volume on MACE risk

**Figure 3:**
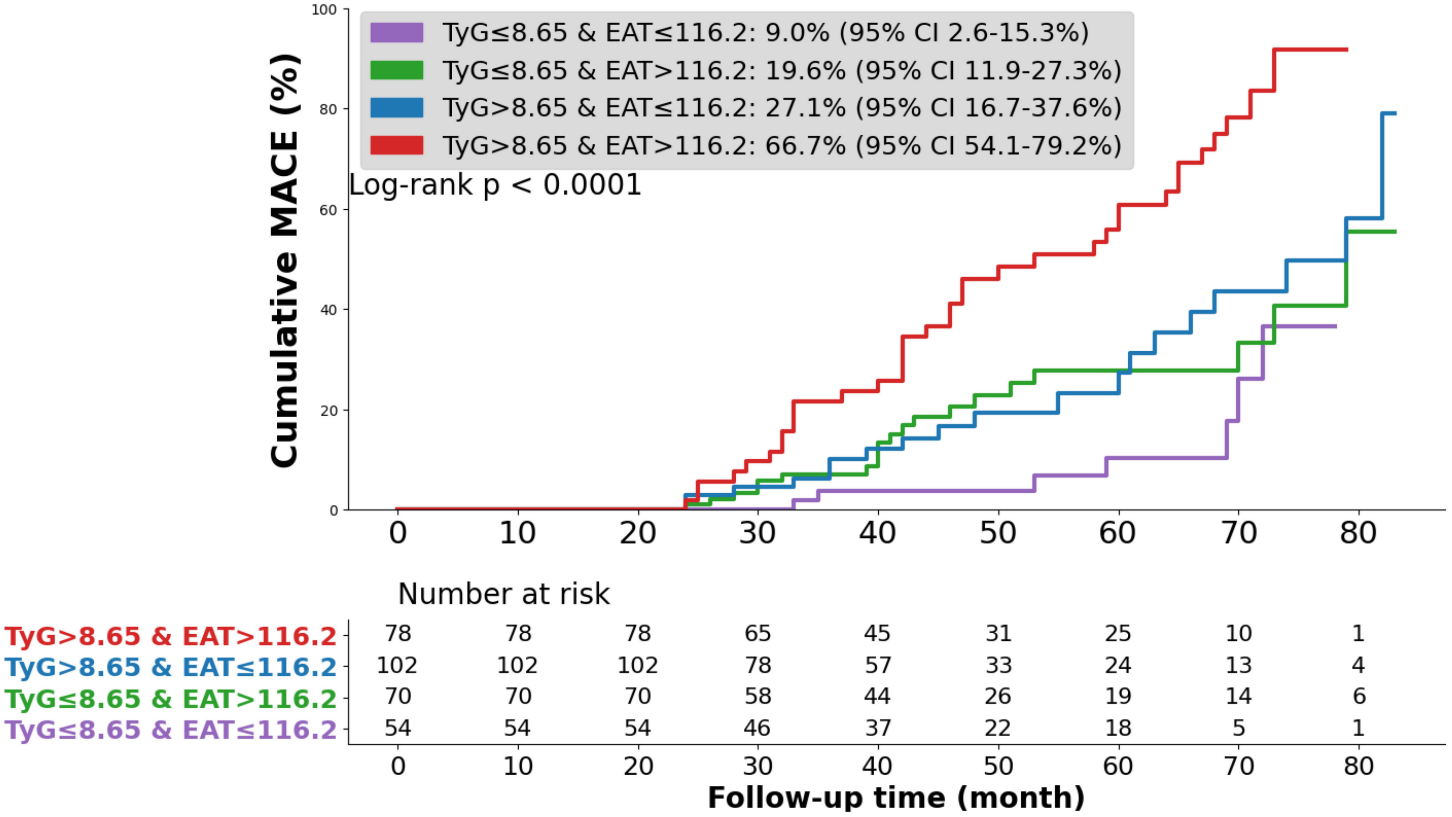
Kaplan–Meier survival curves across TyG index and EAT. TyG index triglyceride-glucose index, EAT epicardial adipose tissue, MACE major adverse cardiovascular event.

Patients were stratified into four groups based on TyG (cut-off 8.65) and EAT volume (cut-off 116.2 cm³). Kaplan–Meier curves showed the highest cumulative MACE incidence in the high TyG/high EAT group and the lowest in the low TyG/low EAT group (*p* < 0.0001, **Figure 3**.).

Univariate Cox regression, high TyG/high EAT was associated with the greatest MACE risk (HR 8.39, 95% CI 3.68–19.10, *p* < 0.001). This remained significant after adjustment for confounders. In the fully adjusted model, the high TyG/high EAT group had an adjusted HR of 7.62 (95% CI 3.27–17.76) compared with the low TyG/low EAT reference group (**Table 4**).

**Table 4:** Joint association of TyG index and EAT with MACE.

TyG index–EAT category	Univariate regression		Multivariate regression*	
	HR (95% CI)	*P*-value	HR (95% CI)	*P*-value
TyG ≤ 8.65 and EAT ≤ 116.2	1 (Ref)		1 (Ref)	
TyG ≤8.65 and EAT>116.2	2.62 (1.11–6.22)	**0.028**	2.28 (0.96–5.42)	**0.045**
TyG>8.65 and EAT ≤ 116.2	3.18 (1.37–7.39)	**0.007**	2.51 (1.03–6.10)	**0.043**
TyG>8.65 and EAT>116.2	8.39 (3.68–19.10)	**<0.001**	6.63 (2.81–15.62)	**<0.001**

TyG index triglyceride-glucose index, EAT epicardial adipose tissue, MACE major adverse cardiovascular events, Ref = reference (baseline) model.

*p* values in bold are < 0.05.

*Adjusted for all the variables in Model 2 plus age, gender, left main disease, triglyceride and serum creatinine.

### 4.4 Interaction between TyG index and EAT volume

As shown in **Table 5**, the combined effect of elevated TyG and increased EAT volume on MACE risk exceeded the sum of their individual effects. The relative excess risk due to interaction (RERI) was 3.81, the attributable proportion (AP) was 0.50, and the synergy index (SI) was 2.34, all statistically significant (*p* < 0.05). These values indicate that 50% of the excess MACE risk in patients with both risk factors could be attributed to their interaction, and that the combined presence of high TyG and high EAT more than doubled the expected risk if acting independently.

**Table 5:** Additive interaction between the TyG index and EAT.

Interaction measure	Value	95% CI	*P*-value
RERI	3.81	0.20–7.42	**0.038**
AP	0.50	0.22–0.78	**<0.001**
SI	2.34	1.06–5.15	**0.020**

TyG index triglyceride-glucose index, EAT epicardial adipose tissue, RERI relative excess risk due to interaction, AP attributable proportion, SI synergy index.

*p* values in bold are < 0.05.

### 4.5 Incremental predictive value of TyG index and EAT volume

As shown in **Table 6**, adding either TyG index or EAT volume individually to the baseline model moderately improved predictive performance. When both TyG and EAT were included simultaneously, the C-statistic increased, indicating improved discrimination for identifying patients at risk of MACE. Consistently, the NRI showed enhanced risk reclassification, and the IDI demonstrated a significant improvement in the model’s overall predictive accuracy. These results indicate that combined use of TyG index and EAT volume provides substantial incremental predictive value for MACE risk assessment.

**Table 6:** The incremental predictive value of the TyG index and EAT for MACE.

Model	C-Statistic (95% CI)	*P*-value	Continuous NRI (95% CI)	*P*-value	IDI (95% CI)	*P*-value
Model3 without TyG & EAT	0.684 (0.592–0.717)	**0.043**	Ref		Ref	
Model 3 + TyG	0.679 (0.592–0.717)	**0.035**	0.315 (0.204–0.421)	**<0.001**	0.266 (0.163–0.369)	**0.001**
Model 3 + EAT	0.697 (0.592–0.717)	**0.010**	0.330 (0.211–0.454)	**<0.001**	0.522 (0.401–0.644)	**<0.001**
Model 3 + TyG + EAT	0.701 (0.5920.717)	**0.004**	0.301 (0.184–0.415)	**<0.001**	0.804 (0.622–0.992)	**0.019**

TyG index triglyceride-glucose index, EAT epicardial adipose tissue, MACE major adverse cardiovascular events, NRI net reclassification improvement, IDI integrated discrimination improvement, Ref = reference (baseline) model.

*p* values in bold are < 0.05.

The C-statistic, NRI, and IDI values were calculated using bootstrap resampling.

### 4.6 Model fit evaluation

As shown in **Table 7**, adding TyG index and EAT volume progressively reduced AIC and BIC, indicating improved model fit. χ² values increased, further supporting better data fitting. All *p*-values were < 0.05, confirming that the model including both variables outperformed the baseline model.

**Table 7:** Assessment of the goodness-of-fit of models.

Model fit statistics	Model3 without TyG & EAT	Model3 + TyG	Model3 + EAT	Model3 + TyG + EAT
AIC	786.78	785.22	783.01	780.41
BIC	818.07	818.92	816.71	816.52
χ^2^	Ref	3.05	6.84	10.63
df	Ref	1	1	2
*p*-value	Ref	0.080	**0.008**	**0.004**

TyG index triglyceride-glucose index, EAT epicardial adipose tissue, AIC Akaike information criterion, BIC Bayesian information criterion, Ref, reference (baseline) model, χ² from likelihood ratio tests comparing models to baseline (Model 3 without TyG & EAT); df equals the number of additional parameters.

*p* values in bold are < 0.05.

## 5 Discussion

### 5.1 Interpretation of findings

#### 5.1.1 Role of the TyG index

Insulin resistance (IR) is central to multiple metabolic abnormalities beyond diabetes, including obesity, hypertension, dyslipidemia—particularly hypertriglyceridemia with low HDL-C—and other features of metabolic syndrome (MetS) (26, 27). The TyG index is increasingly recognized as a reliable surrogate marker for IR and a strong predictor of cardiovascular (CV) morbidity and mortality across different populations (28–30).

#### 5.1.2 Role of EAT

EAT is an independent risk factor for cardiovascular events (31). Owing to its close anatomical proximity to the myocardium and coronary arteries, EAT exerts paracrine and vasocrine effects that influence myocardial function and coronary atherosclerosis (32). Beyond serving as an energy reservoir, EAT is metabolically active and secretes a range of pro-inflammatory cytokines, contributing to IR and chronic inflammation (33). Unlike BMI, which cannot capture visceral adiposity, EAT provides a more direct and specific assessment of cardiometabolic risk, explaining why individuals with similar BMI may still differ substantially in CV risk profiles (34).

#### 5.1.3 Additive effects of the TyG index and EAT

EAT and IR are positively correlated (35). EAT promotes free fatty acid release, ectopic fat deposition, and adipocyte hypertrophy via the Randle cycle, while producing inflammatory mediators such as leptin, lipocalin, TNF-α, and interleukins (IL-1β, IL-6, IL-8, IL-10) (36, 37). Conversely, IR promotes EAT expansion through chronic hyperinsulinemia, which enhances fatty acid and triglyceride synthesis and accelerates adipose tissue accumulation (38, 39).

This bidirectional relationship may contribute to a potential metabolic vicious cycle, thereby influencing cardiovascular risk. In our study, the combination of the TyG index and EAT volume provided modest incremental improvement in identifying high-risk post-CABG patients compared with either marker alone, supporting their additive value in predicting MACE.

### 5.2 Limitations of the model

#### 5.2.1 Measurement of EAT

EAT volume was quantified using a semi-automated protocol in which trained radiologists manually outlined the pericardial contours, after which the AWS workstation automatically computed the epicardial fat volume. Although this approach provides greater consistency than fully manual segmentation, it remains partially operator-dependent and relatively time-consuming, which may limit its applicability in routine clinical settings.

#### 5.2.2 Study sample and data source

This single-center, retrospective study included 304 participants. The relatively limited sample size and number of events reduced statistical power, particularly for interaction analyses between the TyG index and EAT volume. Although multivariable regression models adjusted for potential confounders, the modest number of events and the absence of detailed surgical characteristics—such as graft type, number of grafts, completeness of revascularization, and surgical technique (OPCAB vs. ONCAB)—may affect the robustness and precision of the estimates, as reflected by wide confidence intervals. Consequently, additive interaction analyses (RERI, AP, and SI) should be interpreted cautiously and regarded as exploratory, reflecting potential additive predictive value rather than definitive biological synergism. Cut-off values for the TyG index and EAT volume were derived from ROC analyses within the same cohort; as these thresholds are data-driven, dichotomization may be prone to overfitting and limited external validity. Therefore, ROC-based results should be interpreted as supportive or exploratory, while primary conclusions rely on analyses treating TyG index and EAT volume as continuous variables. In addition, diabetes and hyperlipidemia were defined according to diagnostic standards at the time of data collection, and more recent criteria (e.g., ADA-recommended HbA1c ≥6.5% and updated LDL-C thresholds) were not applied, which may limit comparability with current guidelines. Larger, prospective, multi-center studies are warranted to validate the TyG–EAT model, improve estimate stability, and enhance generalizability across populations.

#### 5.2.3 Conflicting evidence

Although many studies link EAT to CAD, some report opposite findings or provide insufficient evidence. Le Jemtel and Sacks et al. suggest that EAT may promote atherosclerosis in obese patients via secretion of proinflammatory factors and recruitment of immune cells. However, it remains unclear whether this effect occurs in obese patients without coronary atherosclerosis. Most existing evidence derives from cross-sectional clinical or translational studies, which are inherently limited. In our study, higher EAT volume was associated with increased MACE risk post-CABG, but causality cannot be inferred, and residual confounding by metabolic status cannot be excluded. More longitudinal studies are needed to clarify the precise role of EAT in CAD (40, 41).

## 6 Conclusion

Both the TyG index and EAT volume demonstrated prognostic value in CABG patients, and their combined assessment showed an additive interaction, which may offer modest improvement in postoperative risk stratification. Integrating these markers may help identify patients at relatively higher long-term cardiovascular risk. However, larger prospective studies are required to validate these findings and assess their potential clinical utility.

## Data Availability

The raw data supporting the conclusions of this article will be made available by the authors, without undue reservation.
