# Supplementary material for: Additive predictive value of triglyceride-glucose index and epicardial adipose tissue volume for major adverse cardiovascular events following coronary artery bypass grafting
Source: Front Endocrinol (Lausanne). 2026 Jan 14;16:1730404. doi: 10.3389/fendo.2025.1730404 (PMC12846971; doi:10.3389/fendo.2025.1730404)

**Supplementary Table S1.** Variance inflation factor (VIF) assessment of candidate variables included in the multivariable Cox regression model

| **Variable** | **VIF** |
| --- | --- |
| Age | 1.157 |
| Gender | 1.452 |
| BMI | 1.145 |
| LVEF | 1.064 |
| Left main disease | 1.057 |
| Multivessel disease | 1.041 |
| Current smoking | 1.455 |
| Current drinking | 1.374 |
| Hypertension | 1.109 |
| DM | 1.357 |
| Hyperlipidemia | 1.105 |
| FPG | 1.350 |
| TG | 1.424 |
| LDL-C | 1.351 |
| HDL-C | 1.507 |
| SCr | 1.124 |
| Antiplatelet drugs | 1.112 |

**Supplementary Figure S1.** Cross-validation curve for LASSO Cox regression


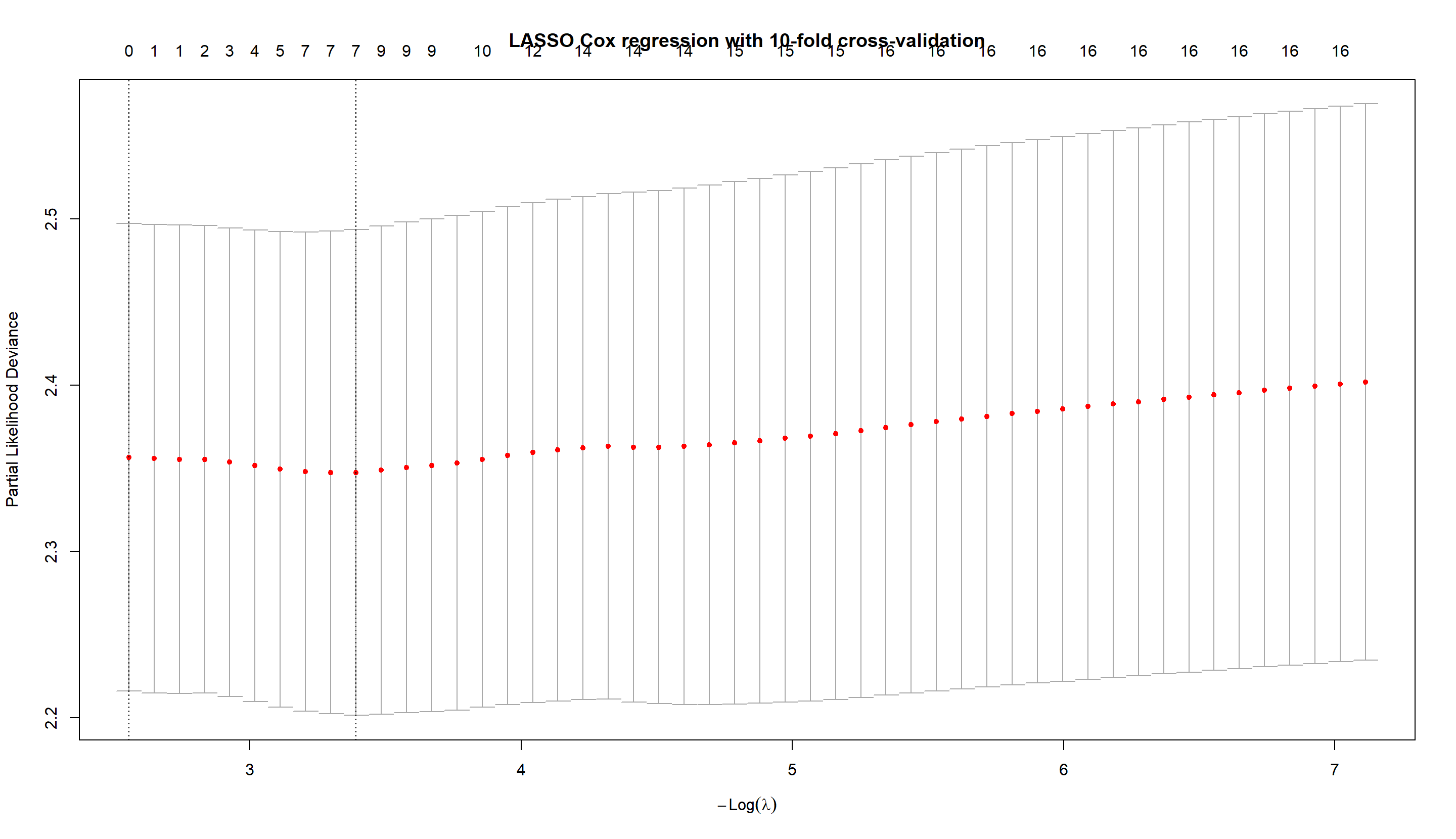

Supplement: Supplementary file 1 [file DataSheet1.docx]
